# Estrogen receptor beta and truncated variants enhance the expression of transfected MMP-1 promoter constructs in response to specific mechanical loading

**DOI:** 10.1186/s13293-014-0014-6

**Published:** 2014-09-27

**Authors:** John D Thaler, Yamini Achari, Ting Lu, Nigel G Shrive, David A Hart

**Affiliations:** 1 McCaig Institute for Bone and Joint Health, University of Calgary, 3330 Hospital Drive NW, Calgary T2N 4 N1, AB, Canada; 2 Schulich School of Engineering, University of Calgary, 2500 University Drive NW, Calgary T2N 1 N4, AB, Canada

**Keywords:** MMP-1, 1G variant, 2G variant, ER-β, Truncated ER-beta, Shear stress and HIG-82 cells

## Abstract

**Background:**

Joint diseases such as osteoarthritis (OA) predominantly afflict post-menopausal women, suggesting a pertinent role for female hormones. Estrogen receptor beta (ER-β) has been detected in connective tissues of the knee joint suggesting that these tissues are responsive to the hormone estrogen. Matrix metalloproteinase-1 (MMP-1) activity contributes to cartilage degradation, a key factor leading to OA development in synovial joints. Two polymorphic forms of MMP-1 exist due to a deletion/insertion of the guanine residue in the promoter, and the 2G allelic variant of MMP-1 exhibits more activity than the 1G allele. Previous studies have demonstrated that the polymorphic forms of the human MMP-1 are influenced by the modulating effects of estrogen receptor isoforms. In addition to hormonal influences, physiological factors such as altered mechanical loading are also contributory features of OA. In the present study, the combined influence of biomechanical and hormonal variables on the activity of MMP-1 isoforms was evaluated. We hypothesized that the combined effects of ER-β and sheer stress will differentially activate the two allelic forms of MMP-1 in a hormone-independent manner.

**Methods:**

HIG-82 synoviocytes were transiently transfected with 1G or 2G alleles (±) ER-β and subjected to either shear or equibiaxial stress. Next, 1G/2G promoter activity was measured to determine the combined influence of physiological stimuli. Truncated ER-β constructs were used to determine the importance of different domains of ER-β on 1G/2G activation.

**Results:**

The 2G allele exhibited a constitutively higher activity than the 1G allele, which was further increased when the transfected cells were subject to shear stress, but not equibiaxial stress. Moreover, the combination of ER-β and shear stress further increased the activity levels of the 1G/2G allelic variants. Additionally, select AF-2 truncated ER-β variants led to increased activity levels for the 2G allele, indicating the AF-1 domain was likely involved in the response to mechanical stimulation.

**Conclusions:**

These results suggest that the 1G/2G alleles of MMP-1 are influenced by specific mechanical stimuli like shear stress, as well as the ER-β receptor. These findings contribute to the potential allelic involvement in connective tissue diseases such as OA in females compared to males.

## 1 Background

Gender differences play a pivotal role not only in the development of chronic diseases such as osteoarthritis and osteoporosis but also in the incidence of knee injuries. It is well recognized that females experience a higher incidence of joint injuries such as tearing of the anterior cruciate ligament of the knee joint than males [1]. Several studies investigating the functioning of the knee have reported alterations in knee laxity and related factors during the menstrual cycle [2],[3]. Studies have implicated hormones and their receptors in the regulation of connective tissues. Evidence for the presence of estrogen and progesterone receptors in the connective tissues of the knee joint has been provided [4],[5], suggesting that these tissues are responsive to hormones which in turn may influence connective tissue turnover. The increased prevalence of osteoarthritis (OA) in women suggests that to understand the sex differences in the development of OA, it is important to examine sex hormone mechanisms in cells from knee tissues [6].

Members of the family of matrix metalloproteinases (MMPs) have been shown to be involved in the catabolic degradation of the connective tissues in the knee joint [7]-[10] and others. One of the MMPs that has been implicated to play a role in the injured connective tissues and degenerative OA is matrix metalloproteinase-1 (MMP-1). MMP-1 is the most ubiquitously expressed collagenase, with most normal connective tissue cells expressing low constitutive levels [11],[12]. However, inflammatory cytokines such as interleukin-1 beta (IL-1β) and tumor necrosis factor-alpha (TNF-α) can stimulate the production of this proteinase. The response of the MMP-1 gene to different stimuli is regulated by the promoter elements in the gene. The transcription factor binding sites located in the promoter region of the MMP-1 gene are essential for understanding the control of gene expression in response to different stimuli. A single nucleotide polymorphism (SNP) has been identified in the promoter of the MMP-1 gene which strongly impacts the response of this gene to different stimuli [13],[14]. This SNP in the promoter of MMP-1 exists at position −1,607 bp, where an additional G residue creates an Ets binding site (5′GGAT3′ compared to 5′GAT3′) adjacent to an AP-1 site at −1,602 bp. The addition of this guanine (G) residue creates a 2G allele which displays greater transcriptional activity than the 1G allele [12]. The presence of a MMP-1 1G/2G polymorphism in the promoter region of the MMP-1 gene is positively correlated with an increased risk for developing various types of cancer such as lung or ovarian cancer [15]. Furthermore, a recent study has also demonstrated that the 1G/2G polymorphism of MMP-1 might be a risk factor for knee osteoarthritis susceptibility in the Greek population [16]. This is in contrast to a separate study which demonstrated an association between the 1G allele and knee OA in a Turkish population [17] and, furthermore, is possibly contrary to expectations that the 2G allele is associated with higher levels of MMP-1 and hence would be associated with development and progression of OA.

Although it is well recognized that the 2G SNP of MMP-1 leads to a higher constitutive activity than the 1G allelic form, the activity of this gene is further influenced by other modulators such retinoids, glucocorticoids, growth factors, sex hormones and their receptors, and mechanical stress. In a previous report, Achari et al. [18] have examined the interaction between estrogen, estrogen receptors (ERs), and the MMP-1 promoter SNP. They reported that MMP-1 promoter variants exhibit differential responses to the ER isoforms α and β. As indicated before, the 2G promoter exhibited higher (~2.5-fold) activity than the 1G variant regardless of the presence of ERs and estrogen. However, the proportional increase in 2G activity in the presence of the estrogen receptor β (ER-β) isoform was less than that of the 1G variant. This study [18] was performed in the absence of mechanical loading which is a common feature of nearly all connective tissue cells in the body.

Mechanical loading is essential for maintaining homeostasis in connective tissues such as those of the knee joint. Connective tissues such as bone, ligaments, cartilage, and menisci consist of unique cell populations in their matrix that can adapt to changing mechanical environments. These tissues are in a continual state of dynamic flux as they experience mechanical input from ground reaction forces, gravity, barometric pressure, vibration, musculoskeletal movement, and contact with external objects [19]. Mechanotransduction, the conversion of mechanical stress (tensile, compression, shear, etc.) into cellular signals, is a well-accepted and a significant response mechanism in bone and other tissues [20]. Cells can also detect external mechanical signals through stretch-activated ion channels as the plasma membrane is deformed [21]. In this manner, mechanical stress applied to the cells can lead to changes in gene expression, which in turn can regulate the physiology of the tissue. Reportedly, many genes including MMP-1 are naturally repressed by joint loading and removal of joint loads can lead to a derepression of genes coding for catabolic mediators in menisci [22]. In addition, this pattern has also been observed in *ex vivo* rat tail tendon experiments. Load-deprived rat tail tendons exhibit a marked increase in pro-MMP-1 and MMP-1 protein production compared to time-zero controls, but when tails were subjected to static tensile loading, there was inhibition of the MMP-1 upregulation [23].

From the studies described above, it is evident that the 1G/2G SNP in the MMP-1 gene can lead to differential responses to physiological stimuli. In the present studies, the combined impact of sex hormone receptor and mechanical stimuli on MMP-1 promoter activity was investigated. The studies were based on the hypothesis that mechanical stimuli will lead to controlled expression of the variants in the MMP-1 promoter in the presence of ligand-independent stimulation by the beta isoform of estrogen receptor (ER-β). Thus, the inheritance of specific MMP-1 promoter variants could be a genetic risk factor for sex-hormone dependent connective tissue conditions or for musculoskeletal degeneration in sedentary or bed-ridden patients, or astronauts exposed to microgravity. Thus, MMP-1 may be regulated very differently in pre-menopausal and post-menopausal females depending on the variations in the hormone levels.

## 2 Methods

### 2.1 Cell culture

The rabbit synoviocyte cell line HIG-82 used in this study was previously shown to be negative for endogenous estrogen receptor alpha (ER-α) and ER-β expression [24],[25] and was obtained from the American Type Culture Collection (Rockville, MD, USA). The cells were cultured in medium consisting of Ham’s F-12 Nutrient Mixture (GIBCO Invitrogen, Carlsbad, CA, USA) supplemented with 10% fetal bovine serum (GIBCO Invitrogen) and 1% antibiotic/antimycotic (GIBCO Invitrogen) in a 5% CO_2_ humidified air chamber at 37°C. The cells were passaged 1:4 with 0.25% trypsin when 80% confluent.

For use in the Flexcell Streamer™ system (Flexcell International Corporation, Hillsborough, NC, USA), the HIG-82 cells were grown in monolayer culture on 72 mm × 25 mm microscope slides. Following sterilization, a set of three slides was placed in one square Petri plate (BD Falcon, San Jose, CA, USA). HIG-82 cells were collected, counted, and seeded on the slides at a density of ~2.5 × 10^3^ cells/cm^2^. For a single experiment, two pairs of plates were designated as either shear exposure or non-shear controls. The cells were cultured in a humidified chamber with 5% CO_2_ at 37°C for 48 h to allow time for the cells to attach onto the slides and reach approximately 80% confluency.

For tensile loading, HIG-82 cells were seeded on the central area of collagen type I coated Bioflex® 6-well cell culture plates (Randolph, NJ, USA) corresponding to the area of uniform strain for biaxial loading [26]. This yielded an approximate density of ~2.5 × 10^3^ cells/cm^2^. Cell cultures were maintained in complete growth medium at 37°C under 5% CO_2_ until approximately 80% confluent. Complete growth medium was replaced with transfection medium 24 h prior to loading. This protocol was adapted from [24] and [27].

### 2.2 Sub-cloning of human MMP-1 promoter constructs

The human 1G and 2G MMP-1 promoter constructs were a generous gift from Dr. C.E Brinckerhoff (Dartmouth Medical School, NH, USA). Both the 1G and 2G constructs were sub-cloned into the pGL-3 luciferase expression vector as previously described by Achari et al. [18].

### 2.3 Additional control and expression plasmids

The expression vector for ER-β was a generous gift from Dr. Koen Dechering (Organon, Oss, the Netherlands). The ER-β insert was subsequently cleaved from the original vector and sub-cloned into the pSG5 vector with orientation and integrity of inserts confirmed by sequencing [25]. The pRLSV40 plasmid (Promega Corp., Madison, WI, USA) was used as an internal control in the dual luciferase assay. The pRLSV40 plasmid constitutively expresses the *Renilla reniformis* form of luciferase under the strong SV40 promoter. The ER-β ABCDE and ER-β ABCD constructs were derived from the full length ER-β construct and sub-cloned into the pSG5 vector (Stratagene, Santa Clara, CA, USA). The sequence and orientation of the sub-cloned fragments were confirmed by sequencing.

### 2.4 Transient transfection

The following protocols for HIG-82 transfection with FuGENE 6 were adapted from those described by Kydd et al. [27] and Lu et al. [24]. HIG-82 cells were transfected with either pGL3-1G or pGL3-2G at a concentration of 0.5 μg/ml using the FuGene6 transfection reagent (Roche Molecular, Indianapolis, IN, USA) as directed by the manufacturer. In addition, the HIG-82 cells were co-transfected with pRLSV40 at a concentration of 0.05 μg/ml which acted as an internal control for the Dual Luciferase Assay System. To evaluate the expression of ER-β, cultures were also co-transfected with pSG5-ERβ or the ER-β variant expression plasmids at a concentration of 0.5 μg/ml to create ER-specific positive cells. We previously determined the 0.5 μg/ml to be the ideal concentration of ER-β for these experiments through an experiment using different concentrations of ER-β ranging from 0.1 to 4 μg [25]. The concentration of 0.5 μg/ml led to readily detectable levels without being overtly overexpressed [25]. For the duration of transfection, Ham’s F-12 medium was supplemented with 1× serum replacement medium (SRM). Plasmid DNA was incubated with FuGENE 6 in 1–2 ml of this medium for 30 min prior to being added to the transfection culture medium to allow for DNA to form complexes with FuGENE 6.

The medium was removed by aspiration from the Petri plates, and the slides were rinsed with 5 ml of 1× phosphate-buffered saline (PBS). The PBS was removed by aspiration and replaced with 10 ml SRM, and 300 μl of transfection mixture was added dropwise to each plate. The plates were incubated in air with 5% CO2 at 37°C for 3 or 6 h. After transfection, the loading regimen was performed.

### 2.5 Loading regimen

The pattern of loading regimen is briefly described below and follows the previously published loading pattern [13],[28]-[32]. It was designed to represent physiological conditions, where loading events are episodic. The amplitudes of mechanical loading chosen are comparable to recent studies [29]-[34] and the frequency of loading was selected to match the stride frequency of a slow 1-m/s walk [35].

#### 2.5.1 Fluid flow-induced shear stress

**Figure 1:**
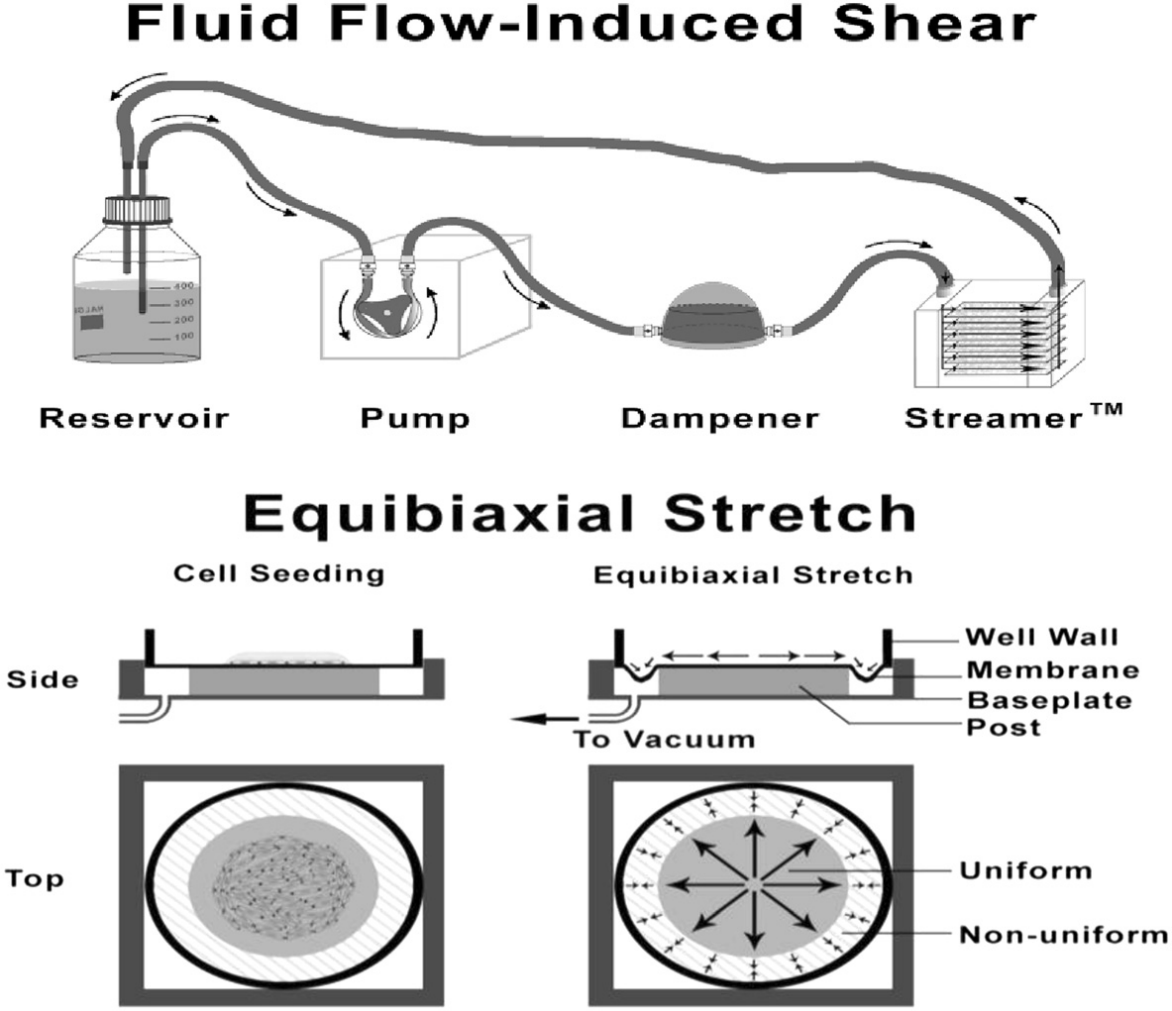
The two modes of loading, sheer and equibiaxial stretch, were applied using two commercial apparatus (top and bottom).

Shear stress was applied using the Flexcell Streamer™ parallel plate flow chamber system (Figure 1) from Flexcell International Corporation. The apparatus was placed in an incubator 30 min prior to and for the duration of the experimental run and maintained at 37°C under 5% CO_2_. The cell culture slides were inserted into the Streamer™ unit. The loading regime consisted of 60 s pulsatile flow oscillating between 10 and 0.8 dyn/cm^2^ at 0.5 Hz followed by 14 min at 0.8 dyn/cm^2^. The 15-min cycle was repeated for 8 h.

#### 2.5.2 Equibiaxial stretch

The cells were exposed to equibiaxial stretch using the Flexcell Tension Plus™ system (Figure 1) contained in an incubator maintained at 37°C under 5% CO_2_. The loading regime consisted of 60 s of EBS oscillating between 2% and 9% elongation at a frequency of 0.5 Hz followed by 14 min of static 2% elongation. The 15-min cycle was repeated for 8 h.

### 2.6 Luciferase assay

Immediately following the final rest period of the loading regimes, the cells were washed with PBS and lysed with 1× passive lysis buffer (Promega Corp.). Luciferase activity within cell lysates was assessed using the Dual Luciferease® Reporter Assay System (Promega Corp.) and a Turner TD-20 luminometer.

### 2.7 Statistical analysis

All loading and transfection experiments were performed in triplicate and repeated at least three times. Statistical analysis of the data was performed using multiple comparisons of variance (two-way, three-way ANOVAs) and the Tukey HSD test calculated on STATA 10 software.

## 3 Results

### 3.1 Effect of fluid flow shear stress on MMP-1 promoter activity

**Figure 2:**
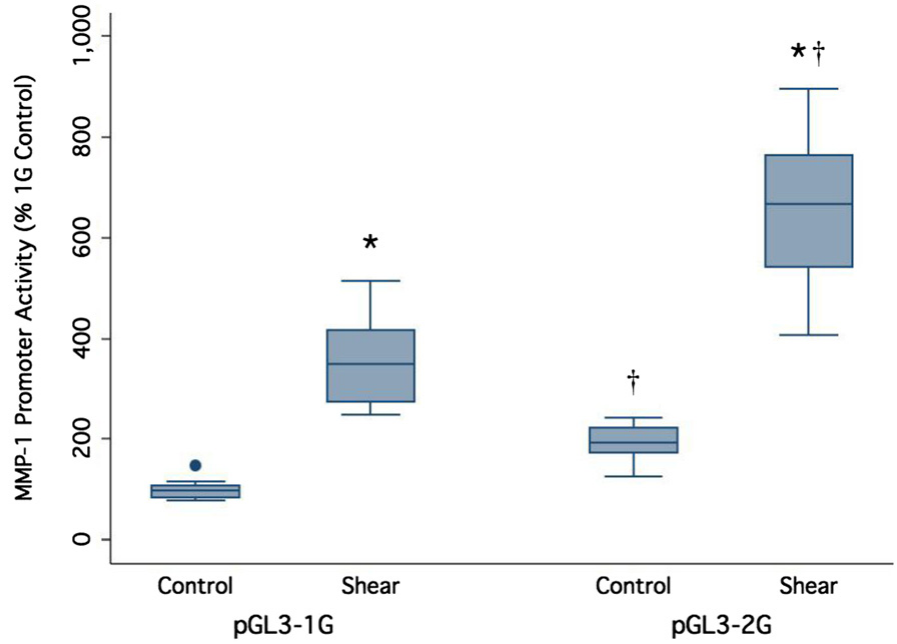
**The effect of fluid flow-induced shear stress on the activity of 1G and 2G MMP-1 single nucleotide polymorphic variants.** *p < 0.05 with respect to matching non-shear control, †*p* < 0.05 with respect to matching 1G variant. Box plots show the median, 25th and 75th percentiles (p25 and p75, lower and upper box boundaries respectively), upper and lower adjacent values (whiskers), and outside values (dots). The inter-quartile range (IQR) is defined as p75–p25. The adjacent values are defined as the highest value not greater than p75 + 3 / 2 IQR and the lowest value not less than p25 − 3 / 2 IQR.

The effect of applying the fluid flow shear stress was tested on the HIG-82 which had been transfected with the 1G and 2G variants of the MMP-1 promoter. The HIG-82 cell line was first transfected with 1G and 2G variants of the MMP-1 promoter. Next, the transfected cells were subjected to 8 h of intermittent pulsating 0.8 to 10 dynes/cm^2^ 0.5 Hz (conditions shown to be optimal in preliminary experiments (data not shown)) for fluid flow-induced shear stress in the flex cell streamer apparatus. Transfected cells placed in the apparatus, not subjected to shear stress were used as controls. A comparison of the 1G and 2G promoters showed that the 2G allele was associated with significantly higher activity than the 1G allele under control conditions (*p* < 0.05) with the 2G promoter activity ~1.8-fold higher than that for the 1G variant, respectively (Figure 2). The promoter activity was significantly increased (~3 fold) for both the 1G and 2G variants of the MMP-1 promoters when transfected cells were subjected to the shear stress protocol (*p* < 0.05) (Figure 2). These results suggest that shear stress enhances the promoter activity of the MMP-1 gene and further suggests that shear stress exposure leads to greater effects on the 2G promoter variant of the MMP-1 gene.

### 3.2 Equibiaxial stretch does not influence MMP-1 promoter activity

**Figure 3:**
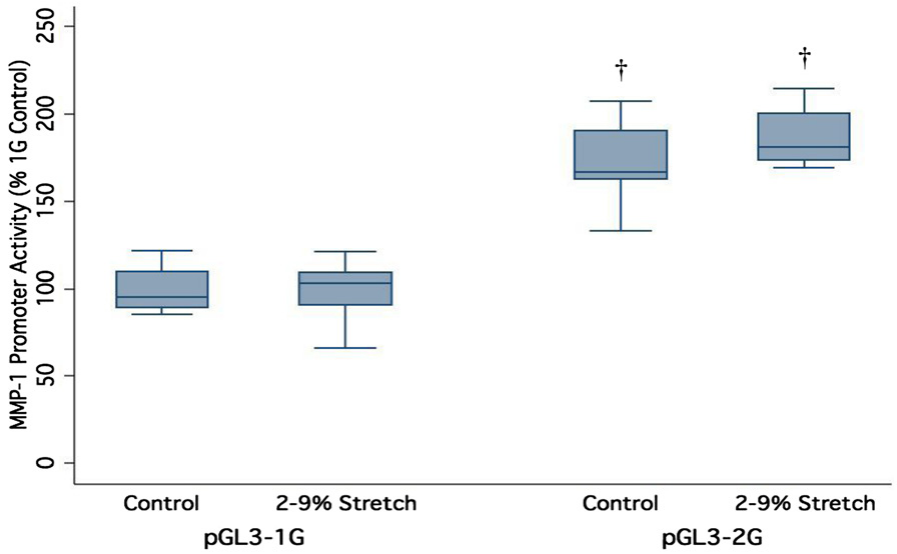
**Effect of intermittent cyclic equibiaxial stretch on MMP-1 promoter activity.** HIG-82 cells were transfected with 1G or 2G promoter-luciferase reporter plasmids and exposed to 8 h of intermittent cyclic 2%–9% 0.5 Hz equibiaxial stretch. Cells were grown on collagen I coated cell culture plates and a subset of plates was subjected to stretch in a Flexcell 4000 system. Unloaded controls were concurrently placed in the stretch apparatus but were not subjected to stretch. MMP-1 promoter activity reported relative to unloaded control 1G promoter activity. (*n* = 9, †*p* < 0.05 wrt matching 1G variant).

The second mode of mechanical stress tested was the effect of cyclic biaxial stretch using the Flexcell Tension Plus system. The HIG-82 cells were exposed to 8 h of intermittent cyclic 2%–9%, 0.5 Hz biaxial stretch on collagen I coated culture plates (Bioflex®). The results depicted in Figure 3 show that MMP-1 promoter activity for either SNP variant was not significantly different compared to the unstretched controls (*p* = 0.3). Typically, the polymorphism of the MMP-1 promoter did affect the constitutive promoter activity of the 1G and 2G alleles for the unloaded and stretched conditions, respectively. There were no significant interactions between the 1G/2G SNP and biaxial stretch exposure (*p* = 0.3).

### 3.3 Role of biological and mechanical stimuli on MMP-1 promoter activity

As described earlier, shear stress has the ability to influence the activity of the MMP-1 promoter. Previously, we have also demonstrated that the presence of the ER isoforms ER-α or ER-β differentially led to increases in the activity of the 1G and 2G MMP-1 promoter variants in HIG-82 cells [18]. In this part of the study, both mechanical stress and ER-β were introduced concurrently in the HIG-82 model system to study their combined effects. The studies focused on ER-β as this isoform is very prevalent in connective tissues.

**Figure 4:**
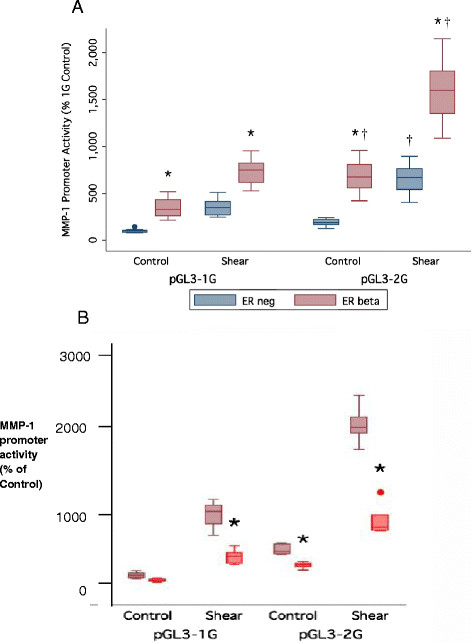
**Combined effect of ERβ and effect of 17β-estradiol. (A)** Combined effect of ERβ expression and shear stress on MMP-1 promoter activity. HIG-82 cells were exposed to 8 h of intermittent pulsatile 0.8–10 dynes/cm 20.5 Hz fluid flow-induced shear stress in a Flexcell Streamer apparatus. MMP-1 promoter activity reported relative to ER negative, unloaded control 1G promoter activity. (*n* = 15, *p < 0.05 with respect to matching ER negative controls, †*p* < 0.05 with respect to matching 1G variant). **(B)** Effect of 17β-estradiol on ER-β-mediated response for MMP-1 promoter activity. HIG-82 cells were transfected with 1G or 2G promoter-luciferase reporter plasmids ± co-transfection with ER-β expression plasmids then exposed to 8 h intermittent pulsatile 0.8–10 dynes/cm2 0.5 Hz fluid flow-induced shear stress in a Flexcell Streamer apparatus with, ±10^−8^ M 17β-estradiol (+shown by orange bars) in medium. MMP-1 promoter activity reported relative to ERβ positive, unloaded control 1G promoter activity. (*n* = 9 except ERβ + Estrogen *n* = 6, *n.s. wrt matching ER negative, 0 M estrogen controls).

HIG-82 cells were transfected with the ER-β expression vector pSG5-ERβ concurrently with the 1G or 2G variant of the MMP-1 promoter constructs. After transfection and 8 h growth in non-shear control conditions, the ER-β expressing cells exhibited MMP-1 promoter activity that was 3.5-fold higher than the ER negative controls (Figure 4A). This is a similar MMP-1 promoter response to ER-β as was reported previously by Achari et al. [18]. Next, the transfected cells were subjected to shear stress conditions. The ER-β transfected cells exhibited a mean 2- and 2.5-fold higher promoter activity than those of the shear-exposed ER negative cells for the 1G and 2G variants, respectively (Figure 4A). In the presence of the 2G allele, shear stress and ER-β also combined to produce a significantly higher MMP-1 promoter activity than in the controls. The effect of ER-β in the absence of estrogen was a two- to threefold increase in promoter activity. Thus, the effects appeared to be additive rather than synergistic.

Interestingly, in the presence of the ligand 17β-Estradiol, the activity of both MMP-1 alleles (1G and 2G) exhibited a proportional decrease (Figure 4B) when ER-β was present. The effect of 10^−8^ M 17β-estradiol, a saturating dose of estradiol [18],[24],[25], on cells expressing ER-β led to promoter activities being depressed 28% to 67% compared to their corresponding control with no hormones. The effect of ER-β in the absence of estrogen was a two to threefold increase in promoter activity; however, in the presence of ligand, the effect of ER-β was largely abrogated (Figure 4B).

### 3.4 Effect of ER-β variants on MMP-1 promoter SNP variant activity under shear stress conditions

**Figure 5:**
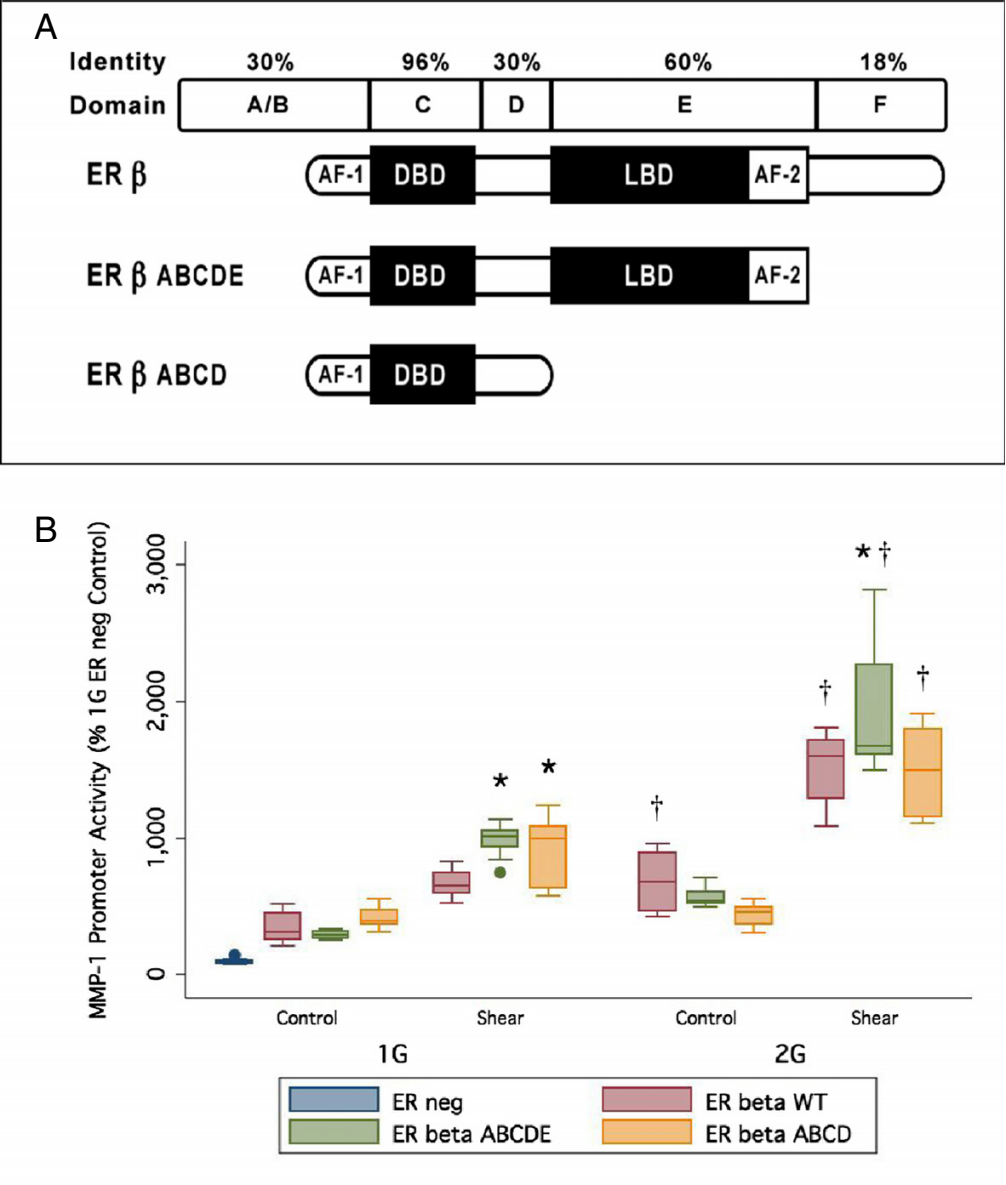
**Classic functional domains and effect of N-terminal ERβ variants. (A)** Classic functional domains of estrogen receptors (ER). **(B)** Effect of N-terminal truncated ERβ variants on MMP-1 promoter activity. AF-1 and AF-2 are activation function regions, DBD is the DNA binding domain, and LBD is the ligand binding domain. ERβ ABCDE and ERβ ABCD are truncated variants lacking the F and E + F domains, respectively. HIG-82 cells were transfected with 1G or 2G promoter-luciferase reporter plasmids ± co-transfection with ER-β variant expression plasmids then exposed to 8 hs intermittent pulsatile 0.8–10 dynes/cm^2^ 0.5 Hz fluid flow-induced shear stress in a Flexcell Streamer apparatus. MMP-1 promoter activity reported relative to ER negative, unloaded control 1G promoter activity. (*n* = 9 except 2G ER-β ABCD *n* = 6, *p < 0.05 wrt WT ER-β, †*p* < 0.05 wrt matching 1G variant).

The ER-β protein contains several specialized domains associated with particular mechanisms by which it influences gene expression, specifically the AF-1 and AF-2 domains [36]. To determine the potential role of the AF-1 and AF-2 domains in influencing the MMP-1 promoter activity, HIG-82 cells were transfected with truncated versions of the ER-β gene (pSG5-ERβ ABCDE and pSG5-ERβ ABCD) (See Figure 5A for a diagram of ER-β domain structure and variants) and either pGL3-1G or pGL3-2G.

Transfected cells were exposed to fluid-flow shear stress as in the previous experiments. When HIG-82 cells transfected with ER-β variants lacking the F domain or the E + F domains were grown for 8 h in non-shear control conditions, there was no significant difference in MMP-1 promoter activity between the full length ER-β and the truncated ER variants (Figure 5B). After 8 h under shear stress conditions, the ER-β ABCDE variant had a positive effect on MMP-1 promoter activity compared to wild-type (WT) ER-β (*p* < 0.05) for both the 1G and 2G promoter alleles. When the statistical effect size was calculated, the effect of the ER-β variants was small. The ER-β ABCD variant showed no significant difference compared to WT ER-β for the 2G MMP-1 promoter allele (*p* > 0.05) (see Figure 5B). Therefore, in this ligand-independent stimulation by ER-β, the removal of domains E and F together has only minor modulatory impact, likely indicating that the response to shear is associated with unique sequences in the AF-1 domain of ER-β.

## 4 Discussion

Hormonal changes and mechanical overuse have also been attributed to influence the degradation of the cartilage in knee, a characteristic feature of OA. The higher prevalence of OA in women than in men suggests that sex differences exist in the development of this disease. Furthermore, post-menopausal women are at higher risk suggesting that hormonal changes may contribute to the development of this disease. Indeed, it is reported that tissues of the knee have receptors for sex hormones and they respond to menopause and menstrual cycle changes [6]. However, how the impact caused by changing hormonal levels on the joint tissues is not clear at the molecular level and the present study focused on this issue.

MMP-1 has been implicated in the degradation of cartilage in the articulating surfaces of the synovial joints such as the knee. The presence of a SNP in the MMP-1 promoter consists of a deletion/insertion (1G/2G) of a guanine nucleotide, and several reports suggest that the 2G allelic form leads to enhanced MMP-1 expression [37],[38]. The present study examined the influence of biomechanical and hormonal factors on the expression of the two allelic forms (1G/2G) of the MMP-1 gene. The results presented in this study of human MMP-1 promoter variants suggests that the 2G allele has the capacity to enhance the response patterns to mechanical stimuli in the absence of estrogen hormone. This suggests that females with 2G allelic form may be at higher risk for the development of this disease when the estrogen levels are low, e.g., menopause.

In this study, we evaluated the response of the 1G or 2G forms of the MMP-1 promoter to mechanotransduction stimuli such as the fluid-flow-induced shear stress and equibiaxial stretch. Of the two types of mechanical influences, only shear stress had the ability to elevate the 1G and 2G MMP-1 promoter activity. Moreover, the 2G form of MMP-1 exhibited a significantly higher (*p* < 0.05) activity level than 1G both constitutively and also under the control of this mechanical stress (Figure 2). This response suggests that the HIG-82 cell line possesses a mechanism for sensing fluid flow shear which triggers a signaling pathway affecting the MMP-1 promoter. These results are in agreement with the extensive work by Sun and Yokota who have shown that lower levels of shear (1–5 dynes/cm2) downregulate MMP-1 expression whereas higher levels of shear (6–20 dynes/cm2) lead to an upregulation of MMP-1 expression in rheumatoid arthritis synoviocytes [33],[39]-[42]. Studies conducted using osteoblasts suggest that activation of the MAPK pathway, an increase in COX-2 expression, and release of prostaglandins are important for cellular mechanotransduction events [43]. Furthermore, this study also suggests that ER-β plays ligand-dependent and ligand-independent roles in mechanical signaling in osteoblasts. However, the mechanism by which the mechanical shear stress is translated into changes in MMP-1 expression is not well understood.

Equibiaxial stretch, a second mode of mechanical stimulation tested in the current study, did not produce a significant change in MMP-1 promoter activity (Figure 3). This was unexpected since stretch has been reported to increase MMP-1 expression in human osteoblasts [44] and is reported to suppress MMP-1 expression in human vascular smooth muscle [45]. Several different substrates were tested including plain plastic loading membranes and ones coated with pronectin (from Flexcell Inc.) (data not included); however, none showed a difference between stretched and non-stretched conditions. Therefore, the specific substrate attachment mediating the mechanotransduction is not a likely explanation for the lack of response. In vascular smooth muscle cells, 4 h of cyclic 25% stretch, but not 7% stretch induced activation of JNK and p38 MAPK [46], but this could have been part of an injury response at this high level of stretch. However, it is possible that the magnitude of stretch used in this study was not sufficient to induce a response. Additionally, the choice of a rabbit cell line as a model system may have had an impact since cyclic stretch loading in a rabbit flexor tendon which inhibited MMP-3 expression, failed to produce a change in MMP-1 expression [47]. The stretch mechanosensory signal cascade in HIG-82 cells, if present, was either not activated by the stretch protocol used in this study or did not interact with the MMP-1 promoter SNP.

Mechanical stimuli are not the only factors known to influence MMP-1 expression. The presence of alpha and beta form of estrogen receptors has been demonstrated in human chondrocytes [48]. The expression of ERs in HIG-82 cells has been shown to alter MMP-1 expression patterns, and the promoter activity response is different for 1G and 2G alleles [18]. The current study found a similar significant interaction between the effects of ER-β expression, sheer stress, and the 1G/2G SNP on MMP-1 promoter activity, supporting the hypothesis that the combination of ER and mechanical stimulation had a additive effects on MMP-1 promoter activity. The reason for these elevation is likely due to the activation of different signaling pathways. When the 1G allele is present, the combined effects of shear and ER-β expression did not lead to the same level of activation as was observed with the 2G allele, where a significant additive interaction between the shear and ER effects were observed. This supports the hypothesis that the presence of the additional ETS site in the 2G allele alters the response pattern of this model cell system to complex combinations of stimuli.

MMP-1 has several proximal promoter elements, but these two distal transcriptional regulatory elements (Ets and AP-1 sites at nt −1,607 and −1,602) appear to play an important role in the stimulation of MMP-1 promoter activity. In addition, a separate study which focused on the role of reactive oxygen species on the elevation of MMP-1 promoter activity also established that site-directed mutagenesis of these Ets and AP-1 sites at nt −1,607 and −1,602 led to significant decreases in the activity of the MMP-1 promoter [49]. For activation of the 2G allele, the ER-β receptor likely acts via a non-classical estrogen response element (ERE)-independent activation pathway since the MMP-1 promoter does not contain an ERE sequence [50]. ERs are known to interact with AP-1 transcription factors, particularly c-Jun, c-Fos, and Fra-1 [51]-[53], and as mentioned above, the AP-1 site at −1,602 immediately adjacent to the −1,607 SNP is known to be an important control point for MMP-1 expression [18]. Since there is no ERE in the MMP-1 promoter and ER-β stimulates the 2G allelic form of MMP-1, ER-β likely contributes to a multi-protein complex at the −1,607 bp AP-1/ETS site. Although the exact mechanisms by which shear and ER are affecting the MMP-1 promoter activity are unknown, it likely involves these ETS and AP-1 sites since the 2G allele is influenced by the interaction between these stimuli.

The binding of 17β-estradiol acts to inhibit the positive effect of ER-β on MMP-1 activity. In light of the fact that estrogen levels drop considerably in post-menopausal women, this could explain at least part of the increased prevalence, incidence, and severity of OA in post-menopausal women [54] as this negative control on MMP-1 expression would be diminished and one could expect a potential elevation in MMP-1 production. Estrogen has also been shown to repress MMP-13 promoter activity in the presence of ER-β [25], and this has already been shown for MMP-1 promoter activity in HIG-82 cells [18] albeit without the additional factor of shear stress. Ligand-dependent ER-β action at AP-1 elements has been shown to be inhibitory [55]. Since ligand binding initiates dimerization of ERs [56], it is possible that ER-β monomers have a positive effect on AP-1 elements in the MMP-1 promoter while ER-β homodimers have a reduced or negative effect.

The effect of two truncated variants of ER-β was tested on the 1G/2G promoter SNPs of MMP-1. In the splice variant labeled ER-β ABCDE, the F domain was removed, and in ER-β ABCD, the E and F domains were removed. In ER-β ABCDE, the C terminal “F” domain, which is reported to impact receptor dimerization and modulate gene transcription in a ligand-specific manner was deleted reviewed in [57]. In ER-β ABCD, the ligand binding domain E and the domain F were deleted. We observed that the activity of 1G or 2G forms of MMP-1 were similar or modestly higher in the presence of the truncated forms of ER-β (ER-β ABCD and ER-β ABCDE) to what was observed with WT ER-β. Moreover, the 2G SNP exhibited somewhat higher activity levels than the corresponding 1G variant in the presence of all truncated ER-β versions and sheer stress (Figure 5). Thus, the responsive elements in the ER-β to stress are likely in the AF-1 domain.

In summary, the results of these studies suggest that the dual influence of mechanotransduction and ER-β leads to an elevation in the activity of the two promoter SNP variants of MMP-1. The 2G SNP of MMP-1 exhibits a higher activity level than the corresponding 1G variant under the influence of these physiological stimuli. In addition, the activation of these physiological stimuli is attenuated by the action of the ligand (17-β estradiol). Interestingly, equibiaxial stretch did not influence the activity levels of 1G and 2G SNPs, thereby suggesting that different types of mechanotransduction may influence different genes differently.

## 5 Conclusions

These findings may also be very relevant to the responsiveness of different connective tissues to changing hormonal levels especially in women and suggest that certain genotypes may mean a higher risk for the development of OA.

## Abbreviations

OA: osteoarthritis

MMP-1: matrix metalloproteinase 1

SNP: single nucleotide polymorphism

1G: allelic variant of MMP-1

2G: allelic variant of MMP-1

HIG-82: rabbit synoviocyte cell line

ER: estrogen receptor

ER-β: estrogen receptor beta

ER-α: estrogen receptor alpha

TNF-α: transforming necrosis factor-alpha

IL-1β: interleukin-1 beta

pGL-3: luciferase expression vector

## Competing interests

The authors declare that they have no competing interests.

## Authors’ contributions

JDT was primarily involved in the data acquisition of data, analysis, and interpretation of data. TL was involved in the designing of the constructs use in this study. She was also involved in data acquisition in the initial design of the study. YA has been involved in drafting the manuscript and revising it critically for important intellectual content. DAH has been involved in the primary conception and design of the study and has played an active role in the revising it critically for important intellectual content. NGS was involved in the critical review of the manuscript and played an active role in the design of the biomechanical aspects of this project. All authors read and approved the final manuscript.
